# Stem Cell-Induced Biobridges as Possible Tools to Aid Neuroreconstruction after CNS Injury

**DOI:** 10.3389/fcell.2017.00051

**Published:** 2017-05-10

**Authors:** Jea Y. Lee, Kaya Xu, Hung Nguyen, Vivian A. Guedes, Cesar V. Borlongan, Sandra A. Acosta

**Affiliations:** Department of Neurosurgery and Brain Repair, Center of Excellence for Aging and Brain Repair, University of South Florida College of MedicineTampa, FL, USA

**Keywords:** trauma, cell transplantation, regenerative medicine, neurogenesis, extracellular matrix

## Abstract

Notch-induced mesenchymal stromal cells (MSCs) mediate a distinct mechanism of repair after brain injury by forming a biobridge that facilitates biodistribution of host cells from a neurogenic niche to the area of injury. We have observed the biobridge in an area between the subventricular zone and the injured cortex using immunohistochemistry and laser capture. Cells in the biobridge express high levels of extracellular matrix metalloproteinases (MMPs), specifically MMP-9, which co-localized with a trail of MSCs graft. The transplanted stem cells then become almost undetectable, being replaced by newly recruited host cells. This stem cell-paved biobridge provides support for distal migration of host cells from the subventricular zone to the site of injury. Biobridge formation by transplanted stem cells seems to have a fundamental role in initiating endogenous repair processes. Two major stem cell-mediated repair mechanisms have been proposed thus far: direct cell replacement by transplanted grafts and bystander effects through the secretion of trophic factors including fibroblast growth factor 2 (FGF-2), epidermal growth factor (EGF), stem cell factor (SCF), erythropoietin, and brain-derived neurotrophic factor (BDNF) among others. This groundbreaking observation of biobridge formation by transplanted stem cells represents a novel mechanism for stem cell mediated brain repair. Future studies on graft-host interaction will likely establish biobridge formation as a fundamental mechanism underlying therapeutic effects of stem cells and contribute to the scientific pursuit of developing safe and efficient therapies not only for traumatic brain injury but also for other neurological disorders. The aim of this review is to hypothetically extend concepts related to the formation of biobridges in other central nervous system disorders.

## Avant garde mechanism of transplanted stem cells for brain repair

Stem cells provide a unique opportunity to understand fundamental cell biology processes and for developing new therapeutic strategies to cure neurological diseases (Yasuhara et al., 2006, 2008; Tajiri et al., 2012). Despite recent progress in the field, mechanisms underlying proven therapeutic effects of stem cells are still poorly understood. Two major schools of discipline can be recognized regarding stem cell mediated brain repair mechanisms that follow degenerative disorders (Borlongan et al., 2004; Pastori et al., 2008; Tajiri et al., 2014). The first supports the concept of cell replacement, where dead or dying cells are directly replaced by transplanted stem cells. The second argues in favor of indirect rescue of the damaged brain parenchyma by transplanted stem cells (bystander effects) through secretion of growth factors (Lee et al., 2007; Redmond et al., 2007).

Stem cells self-renew and differentiate into multiple lineages (Hong et al., 2011). They exist at early developmental stages and also throughout adulthood (Ma et al., 2010), and have been shown to participate in homeostasis regulation (Kim et al., 2011). Furthermore, stem cells are a fundamental component of endogenous repair mechanisms and their transplantation into injured organs is associated with therapeutic benefits (Mazzocchi-Jones et al., 2009; Hargus et al., 2010; Lee et al., 2010; Andres et al., 2011; Borlongan, 2011; Barha et al., 2011; Jaskelioff et al., 2011; Liu et al., 2011; Mezey, 2011; Wang et al., 2011; Yasuda et al., 2011). The adult brain possesses two major stem cell niches: the subventricular zone (SVZ) and the subgranular zone, located the wall of the lateral ventricle and in the dentate gyrus (DG) of the hippocampus (Carlén et al., 2009; Sanai et al., 2011), respectively. Quiescent neural stem cells (NSCs) have been described in other brain regions (Robel et al., 2011). The discovery that stem cells are activated after brain injury represents a landmark finding in the search for effective therapies for brain diseases, and has triggered the exploration of novel promising approaches in regenerative medicine (Yasuhara et al., 2006; Mazzocchi-Jones et al., 2009; Hargus et al., 2010; Lee et al., 2010; Andres et al., 2011; Barha et al., 2011; Borlongan, 2011; Jaskelioff et al., 2011; Liu et al., 2011; Mezey, 2011; Wang et al., 2011; Yasuda et al., 2011; Tajiri et al., 2012). This research allowed for the translation of new stem cell biology concepts into clinical trials designed to treat brain disorders (Pollock et al., 2006; Yasuhara et al., 2009; Seol et al., 2011).

**Figure 1:**
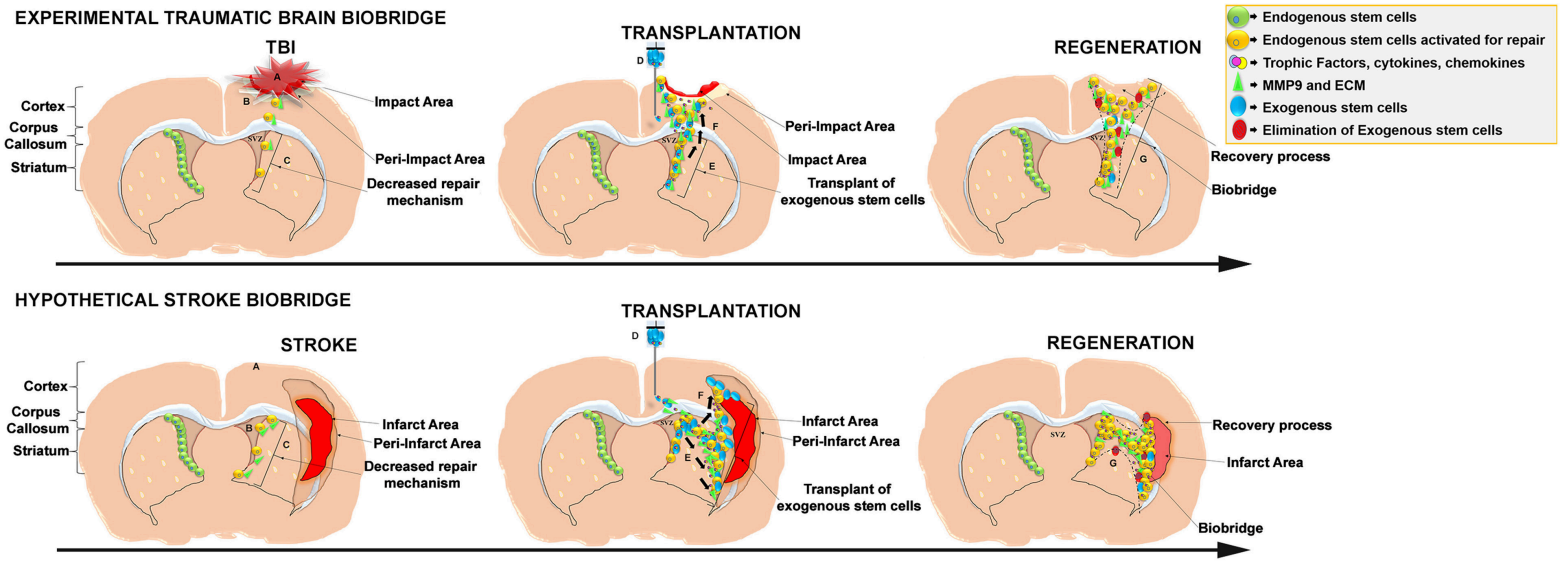
**Schematic representation of the stem cell-mediated brain repair in TBI biobridge from our experimental data and hypothetical stroke biobridge. (Top)** Transplanted stem cells into the injured TBI brain secrete extracellular matrix and metalloproteinases (MMP-9)forming a biobridge between the neurogenic niche (SVZ) and pre-impact area to guide endogenous stem cells to the area of injury. (A), TBI injury; (B), Activation of endogenous repair mechanisms; (C), Decreased repair Mechanism. (D), Transplant of exogenous stem cells; (E) Exogenous stem cells secretion of trophic factors and MMP9 and ECM able to create biobridges of neurovascular matrix; (F), migration of endogenous cells following the biobridge toward the injury site. (G) Elimination of exogenous cells, but maintenance of recovery processes by endogenous stem cells. **(Bottom)** Hypothesized formation of the stroke biobridge, thereby transplanted stem cells may also secrete extracellular matrix and metalloproteinases (MMP-9) contributing to the formation of biobridges and enhancing the migration of endogenous stem cells between the neurogenic niche (SVZ) and peri-infarct area of the cortex and peri-infarct area of the striatum. (A), Stroke injury; (B), Activation of endogenous repair mechanisms; (C), Decreased repair Mechanism. (D), Transplant of exogenous stem cells; (E), Exogenous stem cells secretion of trophic factors and MMP9 and ECM able to create biobridges of neurovascular matrix; (F), Migration of endogenous cells following the biobridge toward the cortex, and striatum (site of injury). (G), Elimination of exogenous cells, but maintenance of recovery processes by endogenous stem cells.

Despite substantial progress in the development of clinically-relevant therapeutic strategies, studies aimed at understanding the mechanisms underlying stem cell-mediated brain repair are still needed to develop safer and more effective therapies. Our group recently showed improvement of traumatic brain injury (TBI) outcomes in rats after intracerebral transplantation of notch-induced human bone marrow-derived mesenchymal stromal cells (referred to as SB623 cells, supplied by SanBio Inc.; Tajiri et al., 2013, 2014; Duncan et al., 2015). Through the investigation of SB623 cells' mechanism of action, our study corroborated the beneficial effects of stem cell transplantation after TBI and provided support for a groundbreaking new stem cell mediated repair mechanism in the brain termed “biobridge.” If was noted that transplanted stem cells can form a “biobridge” connecting the neurogenic niche to the site of brain injury, allowing distal migration of host neurogenic cells and initiation of endogenous repair mechanisms (Tajiri et al., 2014; see Figure 1, Top). In this paper, characteristics and properties of these stem cell paved biobridges are discussed, specifically in regards to the distinct biobridge-mediated mechanism of repair in rats subjected to TBI. Importantly, the clinical significance of this discovery is discussed, and we argue in favor of characterizing this unique stem cell-mediated brain repair mechanism in search of much-needed efficient treatments for neurological disorders and brain injury.

## Formation of “biobridges” in experimental models of TBI by stem cells

After TBI surgery, rats were transplanted intracerebrally with SB623 cells (gene-modified human mesenchymal stromal cells; Zhao et al., 2007; Yasuhara et al., 2009; Tajiri et al., 2014). During the first 3 months, these rats underwent neurological tests to examine the therapeutic effects of SB623 cells transplantation. SB623 cells transplanted rats showed significant improvement in both motor and neurological tests. Moreover, histological assessment revealed a significant reduction in the damage of both the impact and peri-impact area of cortex associated with TBI insult. After transplantation, SB623 cell survival rates were low (0.60 and 0.16% at 1 and 3 months, respectively). Despite the low survival rate of grafted cells, amelioration of both functional behavior and histopathology was achieved. One month after transplantation, it was found a significant increase of Ki67 and nestin, markers of endogenous cellular proliferation and immature neural differentiation, in the peri-injured cortical areas and SVZ. Concurrently, a stream of SB623 cells migrating along the corpus callosum (CC) of transplanted animals was found. At 3 months, SB623 cells transplanted TBI animals showed a significant upregulation of neural differentiation and proliferation markers in the peri-impact cortical area. Nestin and doublecortin (DCX) (immature neurons) were used to identify the migrating cells across the CC from the SVZ to the impacted cortex. Following histological analyses, we examined the formation of the biobridge using laser technology to monitor the cells exiting the SVZ and migrating toward the site of injury. On the other hand, while TBI animals infused with the control vehicle showed increased cellular proliferation, the newly formed cells were limited to the SVZ and cortex (CTX), failing to demonstrate any patterns of migration (Tajiri et al., 2014; Duncan et al., 2015).

## Upregulation of matrix metalloproteinase-9 (MMP-9) and biobridge formation in TBI

The biobridge associating the SVZ and the impacted cortex mainly consisted of vastly proliferative, uncommitted, and migratory cells (Tajiri et al., 2014). Further analysis revealed that animals transplanted with SB623 cells showed a two and nine-fold upregulation of matrix metalloproteinase-9 (MMP-9) activity and expression at 1 and 3 months, respectively (Tajiri et al., 2014). Later, *in vitro* studies demonstrated that SB623 cells enhance migration of endogenous cells via MMP-rich signaling cues. These signals are important for endogenous cell migration and for promoting functional recovery of injured tissue. Only 1 month after TBI, immature Nestin-positive and proliferative Ki67-positive cells were detected in the peri-injured areas and SVZ. Upregulated expression of MMP-9 in the biobridge suggests this neurovascular proteinase is highly important for its formation. Interestingly, this proteinase was upregulated in the control group; however, there was a reversal to sham levels at 3 months post TBI. These results demonstrate the key role of MMP-9 in long-term neural regeneration and functional recovery, accounting for yet another aspect of the action mechanisms through which stem cells intervene during regeneration of damaged brain tissue.

To provide further proof that biobridge formation is accelerated after the transplantation of SB623 cells, an *in vitro* study was performed by co-culturing primary rat cortical neurons and SB623 cells (Tajiri et al., 2014). These cells were cultured in either the presence or absence of the MMP-9 inhibitor Cyclosporin-A (Duncan et al., 2015). It was noted that migration of SB623 cells was improved after primary rat cortical neurons were added. Induced migration was later suppressed by the MMP-9 inhibitor. Although endogenous repair processes begin immediately after TBI, the beneficial effects are limited to the neurogenic SVZ and quiescent neurogenic resident cells surrounding the impacted area. Because, endogenous mechanisms for brain repair are not typically efficient enough to deliver a strong defense against TBI or other disease-induced cell death mechanisms, exogenous cells are transplanted to support the active migration of endogenous stem cells from the neurogenic niche to the site of injury (Tajiri et al., 2014). In the peri-injured cortical areas, stem cell transplants may create a biobridge composed of a neurovascular matrix, allowing newly formed endogenous cells to migrate efficiently to the site of injury. Once the biobridge is established, exogenous cells slowly disappear and are replaced by newly formed endogenous cells that sustain recovery even when the transplanted stem cells are no longer present (Duncan et al., 2015). Of note, previous studies have shown that different cells from notch-induced MSCs from various sources of tissues including umbilical cord blood, peripheral blood (PB), brain can also upregulate the expression of MMP-9 and other extracellular matrix metalloproteinases (Barkho et al., 2008; Sobrino et al., 2012; Lin et al., 2013).

## Injury-specific stem cells migration between the neurogenic niche and the ischemic tissue

Results discussed thus far support the involvement of SB623 cell transplants in the regeneration of the traumatically injured brain through the formation of a biobridge between the SVZ and the peri-injured cortex (Duncan et al., 2015). Formation of a biobridge is a novel mechanism which describes how cell grafts can engage in injury-specific migration between neurogenic and non-neurogenic sites whereby normal cellular motility is restricted. Both *in vitro* and *in vivo* results have shown that transplantation of SB623 cells can improve the histopathological and behavioral deficits associated not only with TBI, but also with stroke, spinal cord injury, and Parkinson's disease (Wang et al., 1996; Tang et al., 1998; Chiang et al., 2001; Failor et al., 2010; Rinholm et al., 2011; Xiong et al., 2011; Merson and Bourne, 2014; Buono et al., 2015; Ranasinghe et al., 2015; Heiss and Zaro-Weber, 2017).

Despite the existence of neurogenic niches in the adult brain, such as the SVZ, that mobilize endogenous cells to repair the stroke brain, a major limiting factor for endogenous repair is the absence of effective cellular migration to the area of injury (Ekdahl et al., 2009; Ducruet et al., 2012; Hassani et al., 2012; Wang et al., 2012; Trueman et al., 2013). Recent findings show that SB623 cell transplantation can improve these endogenous mechanisms by chaperoning new cells from the neurogenic SVZ, through a non-neurogenic area, to the site of injury. The fundamental mechanism of action of SB623 cells therefore is to form biobridges containing MMP-9 and extracellular matrix (ECM), which can lead newly formed cells from the neurogenic area into the ischemic area. In other words, once SB623 cells have successfully constructed these biobridges, endogenous stem cells are facilitated to take part in the regeneration process. As an application of this discovery, the active role of MMP-9 and ECMs in addressing this pathology is identified, which represents an additional therapeutic target for treatment of central nervous system (CNS) injury (Park et al., 2009; del Zoppo et al., 2012). Previously, it has been demonstrated that endogenous MMPs secreted by neural progenitor cells are involved in both the differentiation potential of these cells and with their chemokine-induced cell migration capabilities (Barkho et al., 2008). Interestingly, the expressions of endogenous MMPs specifically MMP-3 and MMP-9 were upregulated after experimental injury to the brain (Barkho et al., 2008). Thus, not only exogenous MMP-9 from grafted cells are important for the migration and proliferation of endogenous neural progenitor cells, but also their own enhanced MMP-9 expression after a molecular or mechanical insult thereby mediating their respond to extrinsic cues (Barkho et al., 2008). Interestingly, it has been demonstrated that various sources of cells including umbilical cord blood, peripheral blood (PB), and the adult brain can influence the functions and levels of MMPs and ECMs (Barkho et al., 2008; Sobrino et al., 2012; Lin et al., 2013), suggesting a potential for MMPs and ECM to as act as biobridge analogs comparable to the present function of Notch-induced SB623 MSCs.

## Graft survival vs. bystander effect

The specific mechanism of action that transplanted SB623 cells employ when integrating into the host tissue and interacting with endogenous stem cells is not well understood. Notably, graft survival is minimal, suggesting the interaction of grafted cells is even more complex. However, low graft survival also indicates that the therapeutic effects of biobridge formation outlast the survival time of SB623 cells. This observation reinforces the notion that endogenous cells begin to play a primary role in the observed therapeutic process after the formation of the biobridge pathway. Inhibition of MMP-9 hindered neurogenic migration from SVZ into damaged tissues. Additionally, after MMP-9 inhibition, there was a remarkable delay in neurovascular regeneration. This experimental result supports the idea that MMP-9 secreted by transplanted cells reinforce the neurovascular unit and induce host-cell migration to the area of injury. However, there is the possibility that cells may wander along the vascular beds. Arguably, the mechanisms might include functional and increased proliferation of the surviving neurons by either cell-to-cell interaction or the bystander effect of secretomes from the stem cells, including cytokines, chemokines, trophic factors, neurotrophic factors, and long non-coding ribonucleic acid (lncRNA) that work as signaling molecules and anti-inflammatory modulators in conjunction with the biobridge to afford neuroprotection and synaptic plasticity (Tajiri et al., 2014). These data have suggested a theory for how the formation of a biobridge could lead to an amelioration of function and recovery after CNS injury.

## Potential therapeutic benefits of biobridge formation in stroke pathology

Adult stem cells are undifferentiated cells that are found throughout the body after early development. In the brain, most of these cells are found in the SVZ of the lateral ventricles and the subgranular zone (SGZ) of the hippocampal dentate gyrus (DG). Adult stem cells generally have limited functions outside of these niches that provide the cells with specific microenvironments wherein stem cells remain in an undifferentiated and self-renewable state. The concept of a niche as a specialized microenvironment housing mammalian stem cells was first put forward by Schofield almost 30 years ago (Schofield, 1978). The niche comprises a basic unit of tissue physiology, integrating signals that mediate the balanced response of stem cells with the needs of the organism. The interaction between stem cells and the more recently documented neurogenic niche must be fully understood if we are to achieve the ultimate goal of designing stem cell therapies targeting CNS injuries.

Our study (Tajiri et al., 2013) proposed the concept of the biobridge mechanism as a cell-mediated repair strategy after TBI. To optimize the translation of this concept to clinical trials of SB623 cells not only in TBI but also in other neurodegenerative disorders, it will be imperative to monitor long-term safety and efficacy of the therapy in animal models. Meanwhile, basic science research will continue in order to gain a more concrete understanding of the stem cell-mediated mechanism of repair in in order to advance the concept of biobridges formation in other neurodegenerative diseases.

Indeed, aside from mechanical brain injury, there are many other neurodegenerative conditions characterized by a “biological gap” between the site of injury and the neurogenic niche that endogenous stem cells have difficulty traversing. Stem cell biobridges facilitate functional remodeling by promoting a series of interactions between endogenous stem cells and damaged tissue by promoting neuronal differentiation, enhancing neural processing, encouraging regeneration of cortical tissue, aiding in intercellular communication, reducing inflammation and scar formation (Tajiri et al., 2013, 2014; Duncan et al., 2015). Thus, a more in-depth understanding of the concept of the stem cell-paved biobridge in neurodegenerative diseases potentially amendable by this therapeutic action, including ischemic stroke, hypoxic-ischemic encephalopathy (HIE), and cerebral palsy (CP) among others, may yield important therapeutic benefits (Tajiri et al., 2014). These disorders are each characterized, in part, by sites of cellular degeneration, separated from neurogenic areas of the brain that could otherwise facilitate the recovery of severe damaged cells (Failor et al., 2010; Rinholm et al., 2011; Xiong et al., 2011; Tajiri et al., 2013; Merson and Bourne, 2014; Tajiri et al., 2014; Duncan et al., 2015; Ranasinghe et al., 2015; Heiss and Zaro-Weber, 2017).

## Biobridge induced-cell migration for neurorestoration in ischemic stroke pathology

Ischemic stroke is characterized by the disruption of blood flow to a region within the brain. Minutes after infarction, areas with the largest decrease of blood flow are irreversibly damaged resulting in the formation of the necrotic core. Areas that are less damaged and still able to maintain metabolic functions are termed ischemic penumbra which surrounds the necrotic core (Taylor et al., 2008; Broughton et al., 2009; Tajiri et al., 2013, 2014; Duncan et al., 2015; Heiss and Zaro-Weber, 2017). Total or partial circulation blockade within the CNS results in cerebral ischemic injury which instigates deprivation of glucose and oxygen resulting in the activation of complex pathological pathways and neuronal cell death (Xiong et al., 2011; Merson and Bourne, 2014; An et al., 2015; Duncan et al., 2015; Bivard et al., 2016). In order to prevent further degeneration and necrosis of the penumbra, blood flow must be re-established within a narrow window on 4.5 h post stroke. Accumulating evidence suggests using tissue plasminogen activator (tPA) therapy within this therapeutic window (4.5 h post stroke) may be beneficial, however, this treatment involves risk and is therefore limited in its application (Graham, 2003; Hess and Borlongan, 2008; Yang et al., 2009; Eissa et al., 2012; Duncan et al., 2015).

In stroke, while the ischemic core cannot be recovered, the penumbra has the potential for repair (Tajiri et al., 2014). Following the finding of the formation of the biobridge in a TBI model, it is not out of contest to think that intracerebral stem cell transplantation within an experimental ischemic stroke model will also result in an equivalent biobridge formation from the neurogenic niche to the infarct area, i.e., cortex and striatum, whereby functional recovery will be facilitated by the promotion of endogenous stem cells migration to the area of injury (see Figure 1, Bottom). To this end, the biobridge has the ability to form a path to the tissue in need of neurorestoration and confer therapeutic benefit in CNS injury (Stone et al., 2013; Tajiri et al., 2013, 2014; Duncan et al., 2015).

The capacity of the biobridge to induce migration of cell across otherwise impenetrable tissues to a damaged area warrants great possibilities for the ischemic tissue. For the biobridge formation in stroke, ECM and MMP-9's secretion and upregulation would be closely characterized to examine the therapeutic benefits of this mechanism and evaluate each of the infarcted areas for markers of neuroregeneration (Yasuhara et al., 2008; Borlongan, 2011). It has been shown that different types of stem cells are able to modify the expression and function of ECM and MMP-9, including stem cells found in umbilical cord, adult brain and PB (Sobrino et al., 2012; Lin et al., 2013). Interestingly, immune modulation is one of the mechanism being studied for the improved functional recovery post-MSC transplantation post ischemic stroke (Yoo et al., 2013). However, the formation of the biobridge is able to further assist by inducing the release of a variety of immunomodulatory and trophic cytokine and chemokines post transplantation (Hsieh et al., 2013). Previously, It has been well established that waves of secreted pro-inflammatory and anti-inflammatory cytokines mediate the immune response throughout the brain and are able to increase the activation and recruitment of immune cells to the area of injury for initial repair (Ekdahl et al., 2009). Indeed, experimental studies will be necessary to confirm the formation of the biobridge and its capability to relocate endogenous stem cells to the site of injury in stroke pathology.

The neurological and histological functional benefits provided by stem cell transplantation through biobridge formation is indicative of the strong connection between endogenous and exogenous repair mechanisms. The beneficial effects of the biobridge mechanism of repair in TBI warrants further investigation in other neurological disorders including stroke. If the formation of this cellular conduit is also efficacious in other neurodegenerative diseases, it could open new venues of potential improvement in the regenerative potential and therapeutic effect of stem cell therapy within the brain of many different neurodegenerative diseases.

Like in TBI, it is possible that a biobridge would form an uninjured brain area to the damaged area post transplantation of stem cells to support endogenous repair mechanisms and function as a neurorestoration mechanism (Tajiri et al., 2013, 2014; Duncan et al., 2015). In order to exploit the therapeutic potential of the biobridge in the treatment of other neurological disorders, additional investigations are necessary to further elucidate the mechanism by which these cells work in conjunction with neurogenic niches to enhance and support regeneration.

## Multi-faceted mechanism involving stem cell therapy

As previously discussed, the main mechanisms of action for stem cells are thought to be either direct cell replacement and/or the release of trophic factors such as FGF-2, EGF, stem cell factor, erythropoietin, and BDNF (Snyder et al., 1997; Borlongan et al., 2004; Lee et al., 2007; Redmond et al., 2007; Pastori et al., 2008; Acosta et al., 2014; Tajiri et al., 2014; Acosta et al., 2015; Duncan et al., 2015). Recently, we proposed a third mechanism in which transplanted stem cells, through the formation of a biobridge, recruit endogenous stem cells from neurogenic niches and mobilize them (Alvarez-Buylla et al., 2008) to the impact and peri-impact areas. One can reasonably hypothesize that the biobridge promotes the migration of endogenous stem cells by releasing migratory trophic factors i.e., cysteine-x-cysteine motif chemokine ligand 14 (CXCL14) and monocyte chemoattractant protein 1 (MCP1) or by constructing a physical “bio-highway” linking the niche to the injured site. Recent studies have suggested that ECM synthesis plays an important role in this process. For example, one study demonstrated that successful migration of interstitial cells depends on scaffold porosity, deformation of the nucleus, and modulators such as pericellular collagenolysis and mechano-coupling (Wolf et al., 2013). Another study revealed that IL-1β induced secretion of trophic factors and adhesion components in the ECM including collagen and laminin, thus enhancing the migration and recruitment of monocytes (Carrero et al., 2012).

To achieve clinically relevant outcomes from stem cell therapy in experimental models of neurodegeneration, it is key to evaluate the fate of recruited endogenous stem cells. Neurogenesis is not necessarily equivalent to the formation of new, integrated neurons in the neural circuit, and it is imperative that further studies characterize the physiology and functionality of new cells *in vivo* to determine their contribution to sustenance or regeneration of damaged neural networks. Moreover, to produce beneficial effects, stem cells must differentiate into the relevant disease-phenotype (Hong et al., 2011), i.e., basal ganglia neurons or substantia nigra neurons for Huntington's disease and Parkinson's disease, respectively (Gantz et al., 2011; Dupuis et al., 2013; Fieblinger et al., 2014; Escande et al., 2016). The biobridge might help newly differentiated stem cells migrate toward the desired area of effect. However, it is important to consider that both transplanted stem cells and endogenous stem cells may differentiate into neurons. If the stem cells do not differentiate into functional neurons, we suggest that the biobridge may also contribute to neurogenesis via the by-stander effect. By directing the flow of stem cells to the injured area, the biobridge may help concentrate growth factors and/or other beneficial molecules such as anti-inflammatory, anti-apoptotic, and anti-oxidative stress reducers.

The concept of the biobridge shares some resemblances with the use of olfactory ensheathing glia in the treatment of spinal cord injury (Tajiri et al., 2014; Duncan et al., 2015). *In vivo* and clinical studies strongly suggest that transplantation of olfactory ensheathing cells (OECs) in combination with specific physical training might provide therapeutic benefits for CNS injuries and neurodegenerative diseases. OECs are capable of producing cell adhesion molecules and growth factors that facilitate the survival of neurons and promote neurite outgrowth (He et al., 2013). Furthermore, transplantation of OECS and Schwann cells (SCs) in a sub-acute phase of spinal cord contusion increased the number of spared/regenerated supraspinal fibers, enhanced tissue integrity, reduced cavitation, and improved overall anatomical outcomes (Barbour et al., 2013). In many spinal cord injury models, the therapeutic outcomes of OEC transplantation have been attributed to the secretion of growth factors, axonal and neuronal regeneration, and/or remyelination (Roet et al., 2013; Tajiri et al., 2014). While there are similarities between the discussed biobridge and the transplantation of OECs in spinal cord injury, there is one major difference between the two therapies. The OECs in the transplantation model were accompanied by artificial scaffolds comprised of laminin and fribronectin. In contrast, the biobridge occurs without any artificial biomaterials as the stem cells remodel matrices and promote the migration of the endogenous stem cells themselves.

An occurrence similar to the biobridge concept was documented in Parkinson's disease studies, where dopamine-secreting cells were transplanted along the nigrostriatial pathway rather than only in the striatum to mimic the natural afferent and efferent dopaminergic pathways (Wang et al., 1996; Tang et al., 1998; Chiang et al., 2001; Tajiri et al., 2014; Duncan et al., 2015). These studies artificially created a bridge between the substantia nigra and the striatum by micro-injections of immature cells along the nigrostriatal pathways. Collectively, these studies suggest that successful stem cell therapy depends on various overlapping treatment processes, mainly through by-stander effect, biobridge formation and to a less extent cell replacement, which work together to promote the observed therapeutic benefits (Tajiri et al., 2013, 2014).

## Author contributions

CB conceptualized this study. JL, KX, HN, VG, CB, and SA wrote the review.

### Conflict of interest statement

CB is an inventor on a patent application related to the stem cell research reported here. CB received research financial support from SanBio Inc. for this study. CB is additionally supported by NIHNINDSR01NS071956-01, James and Esther King Foundation for Biomedical Research Program, Celgene Cellular Therapeutics, KMPHC and Neural Stem Inc. Financial support for this study was through Sanbio Inc. CB is additionally funded by the James and Esther King Biomedical Research Foundation 1KG01-33966, NIH 5U01NS055914-04 and NIH 1R01NS071956-01A1, Celgene Cellular Therapeutics, KMPHC and NeuralStem Inc. The funders had no role in study design, data collection and analysis, decision to publish, or preparation of the manuscript. The other authors declare that the research was conducted in the absence of any commercial or financial relationships that could be construed as a potential conflict of interest.
